# NAD Metabolome Analysis in Human Cells Using ^1^H NMR Spectroscopy

**DOI:** 10.3390/ijms19123906

**Published:** 2018-12-06

**Authors:** Konstantin Shabalin, Kirill Nerinovski, Alexander Yakimov, Veronika Kulikova, Maria Svetlova, Ljudmila Solovjeva, Mikhail Khodorkovskiy, Stepan Gambaryan, Richard Cunningham, Marie E. Migaud, Mathias Ziegler, Andrey Nikiforov

**Affiliations:** 1 Institute of Cytology, Russian Academy of Sciences, St. Petersburg 194064, Russia; konstantin.shabalin@gmail.com (K.S.); nerinovski@yandex.ru (K.N.); veronika.a.kulikova@gmail.com (V.K.); svetlma@mail.ru (M.S.); mila.solovjeva@gmail.com (L.S.); 2 Petersburg Nuclear Physics Institute, NRC Kurchatov Institute, Gatchina 188300, Russia; yaleks@gmail.com; 3 Department of Nuclear Physics Research Methods, St. Petersburg State University, St. Petersburg 199034, Russia; 4 Peter the Great St. Petersburg Polytechnic University, St. Petersburg 195251, Russia; khodorkovskii@gmail.com; 5 Sechenov Institute of Evolutionary Physiology and Biochemistry, Russian Academy of Sciences, St. Petersburg 194223, Russia; gambaryan.stepan@gmail.com; 6 Mitchell Cancer Institute, University of South Alabama, Mobile, AL 36604, USA; rcunningham09@qub.ac.uk (R.C.); mmigaud@health.southalabama.edu (M.E.M.); 7 Department of Biomedicine, University of Bergen, 5020 Bergen, Norway; mathias.ziegler@uib.no

**Keywords:** Vitamin B3, NAD metabolome, NMR spectroscopy, human cells

## Abstract

Nicotinamide adenine dinucleotide (NAD) and its phosphorylated form, NADP, are the major coenzymes of redox reactions in central metabolic pathways. Nicotinamide adenine dinucleotide is also used to generate second messengers, such as cyclic ADP-ribose, and serves as substrate for protein modifications including ADP-ribosylation and protein deacetylation by sirtuins. The regulation of these metabolic and signaling processes depends on NAD availability. Generally, human cells accomplish their NAD supply through biosynthesis using different forms of vitamin B3: Nicotinamide (Nam) and nicotinic acid as well as nicotinamide riboside (NR) and nicotinic acid riboside (NAR). These precursors are converted to the corresponding mononucleotides NMN and NAMN, which are adenylylated to the dinucleotides NAD and NAAD, respectively. Here, we have developed an NMR-based experimental approach to detect and quantify NAD(P) and its biosynthetic intermediates in human cell extracts. Using this method, we have determined NAD, NADP, NMN and Nam pools in HEK293 cells cultivated in standard culture medium containing Nam as the only NAD precursor. When cells were grown in the additional presence of both NAR and NR, intracellular pools of deamidated NAD intermediates (NAR, NAMN and NAAD) were also detectable. We have also tested this method to quantify NAD+ in human platelets and erythrocytes. Our results demonstrate that ^1^H NMR spectroscopy provides a powerful method for the assessment of the cellular NAD metabolome.

## 1. Introduction

**Figure 1:**
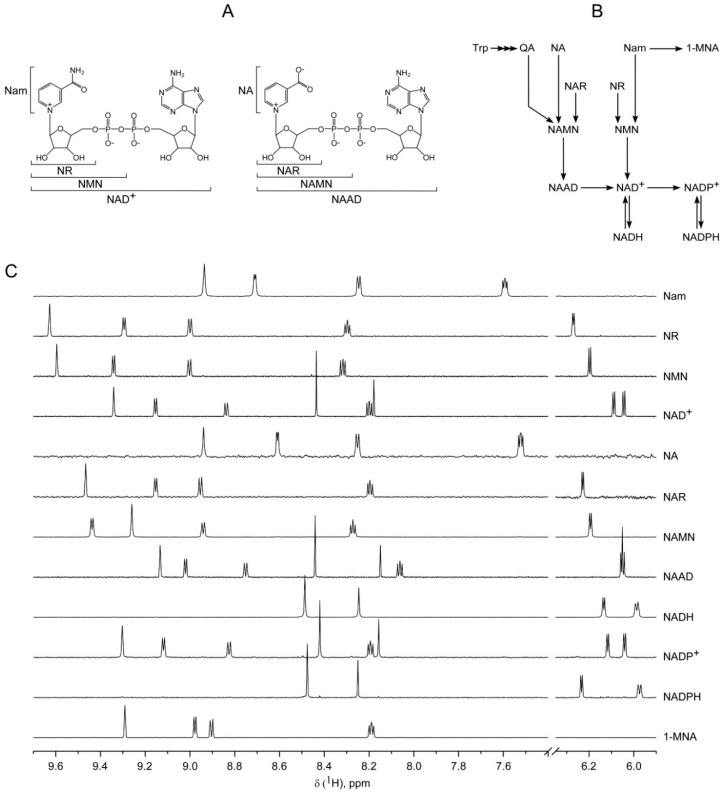
Detection of NAD^+^ and its major intermediates by ^1^H NMR spectroscopy. (**A**) Structures of NAD^+^ and its major intermediates. (**B**) Schematic overview of nicotinamide adenine dinucleotide (phosphate) NAD(P) biosynthetic pathways in humans. (**C**) 700 MHz ^1^H NMR spectra of NAD^+^ and its major intermediates. Only signals used for analysis are shown. Metabolites were dissolved in 50 mM sodium phosphate buffer in D_2_O (pH 6.5). 1 mM sucrose was used as a chemical shift reference (*δ*(1H), 5.42 ppm) and internal standard for quantification.

Nicotinamide adenine dinucleotide (NAD) and its phosphorylated form, NADP, are the major coenzymes of redox reactions in central metabolic pathways. Besides its crucial role in cellular metabolism, NAD also serves as substrate of several families of regulatory proteins: Protein deacetylases (sirtuins), ADP-ribosyltransferases (ARTs) and poly (ADP-ribose) polymerases (PARPs), which govern vital processes including gene expression, DNA repair, apoptosis, aging, cell cycle progression and many others [1,2,3,4]. Nicotinamide adenine dinucleotide is also used by ADP-ribosyl cyclases (CD38, CD157) to produce calcium mobilizing messengers, including ADP-ribose (ADPR) and cyclic ADP-ribose (cADPR) [5,6]. The regulation of these metabolic and signaling processes depends on NAD availability, that is, a continuous replenishment of cellular NAD pools. Generally, human cells regulate their NAD supply through biosynthesis using tryptophan (Trp) or various forms of vitamin B3: The pyridine bases nicotinamide (Nam) and nicotinic acid (NA) or the nucleosides Nam riboside (NR) and NA riboside (NAR) (Figure 1A). Nicotinamide and NA are converted to the corresponding mononucleotides (NMN and NAMN) by nicotinamide phosphoribosyltransferase (NamPRT) and nicotinic acid phosphoribosyltransferase (NAPRT), respectively. Nicotinamide mononucleotide and NAMN are also generated through phosphorylation of NR and NAR, respectively, by nicotinamide riboside kinases (NRK). Nicotinic acid mononucleotide and NMN are converted to the corresponding dinucleotides (NAAD or NAD^+^) by NMN adenylyltransferases (NMNAT). NAD synthetase (NADS) amidates NAAD to NAD^+^ (Figure 1A,B) [4,7].

The measurement not only of NAD^+^/NADH and NADP^+^/NADPH but also their biosynthetic intermediates has gained considerable importance, because vitamin B3 supplementation has been shown to provide strong positive effects in several pathological states, especially aging-associated diseases [8,9]. Measurements of pyridine nucleotides have been complicated, because of their oxidized and reduced states which result in different chemical stabilities. For example, acid extraction, commonly used for nucleotide determinations, is not suitable for NAD(P)H measurements, because reduced pyridine nucleotides are readily degraded under these conditions. Therefore, most methods to determine the NAD metabolome rely on extraction procedures using organic solvents. High-performance liquid chromatography procedures using absorbance for detection lack precision due to low sensitivity and co-eluting contaminants. Therefore, quantification of NAD and its metabolites from biological samples is currently conducted mainly using liquid chromatography coupled to mass spectrometry (LC-MS) [10,11,12,13]. This technology has provided accurate and sensitive measurements in many studies for most intermediates. However, the ionization of some metabolites is rather inefficient. Moreover, the composition of the biological samples affects the measurement rather strongly. Therefore, for accurate quantification, isotope-labeled standard compounds need to be added to the samples. However, for many NAD intermediates, such standards are not commercially available.

These problems and limitations can be overcome using NMR spectroscopy. During the last decades, a number of important technical developments have turned NMR into a powerful analytical technique enabling the identification and quantification of the chemical composition in a wide range of samples. In particular, NMR has become a valuable method in metabolomics, where it is used to study large sets of biological samples. An important advantage of this method is the possibility to directly identify the various detected metabolites, irrespective of the complexity of the sample. Moreover, given its physical principle, NMR represents an intrinsically quantitative method, as the intensity of the registered signal is proportional to the number of resonating nuclei in the sample. That is, the relative concentrations of different components in the sample can be directly inferred from the NMR spectrum. Absolute concentrations are readily determined by comparison to signal intensities obtained from analyses of standard compounds at known concentrations. Thereby, NMR represents one of the most informative techniques in the analysis of biological samples. Consequently, this method is also of interest for the study of the NAD metabolome, as indicated in a few recent studies [14,15,16].

In this study, we have systematically developed an NMR-based experimental approach for the comprehensive analysis of NAD(P) and its major intermediates in human cell extracts. Using this method, we detected and quantified NAD^+^, NADP^+^, NMN and Nam pools in HEK293 cells cultivated in standard culture medium containing Nam as the only available NAD precursor. When cells were grown in the presence of NR and NAR, we additionally identified intracellular pools of deamidated NAD intermediates (NAR, NAMN and NAAD). We have also shown that this method can be used to quantify NAD+ in human blood cells.

## 2. Results and Discussion

### 2.1. Optimization of NMR-Based Quantification of the NAD Metabolome

To achieve the best possible sensitivity and accuracy, we first optimized parameters of NMR spectra acquisition for simultaneous analysis of NAD and its major intermediates. Standard solutions of Nam, NA, NR, NAR, NMN, NAMN, NAD^+^, NAAD, NADP^+^, NADH, NADPH, and 1-MNA were prepared using D_2_O-based buffer containing 50 mM NaPi (pH 6.5) and 1 mM sucrose as a chemical shift reference (*δ*(1H), 5.42 ppm) and internal standard for quantification.

To suppress the residual solvent signal from the exchangeable water molecules during the relaxation delay, we used the PRESAT pulse sequence implemented in the VNMRJ 4.2 software. The PRESAT is preferable for quantitative analysis because it minimizes experimental artifacts arising from nonuniformity of the NMR signal excitation and high signal-to-noise ratio. 

A ^1^H-spectrum for each metabolite of interest was obtained and several characteristic peaks for the identification and quantification of the metabolites were selected. In Figure 1C, the regions of chemical shifts 5.9–6.3 and 7.4–9.7 ppm are shown for the standard ^1^H spectra of Nam, NA, NR, NAR, NMN, NAMN, NAAD, NAD^+^, NADH, NADP^+^, NADPH and 1-MNA. In these areas, each metabolite exhibits a characteristic set of signals permitting an unambiguous assignment of the chemical compound. Moreover, the majority of signals does not overlap, thereby enabling measurements independent of the sample composition and, consequently, appropriate relative quantification.

The longitudinal relaxation time T_1_ for each metabolite signal was measured to maximize the sensitivity and to choose the proper value of the relaxation delay time. To determine the relaxation time, we used 1 mM solutions of the metabolites in DBP buffer (see Materials and Methods section for details) with minimum and maximum delay times of 0.2 and 60 s, respectively. The results are presented in Table 1. The maximal relaxation time (13.7 s) was measured for the Nam singlet signal at *δ* = 8.94 ppm. 

For the quantitative determination of metabolite concentrations, a 5-fold maximum relaxation delay time is required, that is, for the measurement of the NAD metabolome 68 s. With this relaxation delay time, the signal intensities of all metabolites of interest are proportional to their concentrations. However, using this relaxation delay time, two hours (nt = 128) of data acquisition are required to achieve a signal-to-noise ratio of at least 5 for a 2 µM metabolite solution. To increase the signal-to-noise ratio, shorter relaxation delay times were used which led to an increase in the number of repetitions of the pulse sequence per time, but reduced the signal intensity. To obtain the real signal intensities, we introduced the longitudinal relaxation correction coefficient *α_i_* defined according to Equation (2) with a repetition time of 5 s and the relaxation time shown in Table 1.

To determine the sensitivity, we measured a series of dilutions of a standard NAD^+^ solution. We used four signals with chemical shifts of 9.34, 9.15, 8.84 and 8.43 ppm. When recording spectra with a repetition number nt = 512 and a pulse repetition time of 5 s, the limit for quantification is 2 µM with a standard deviation of 10%. The signal-to-noise ratio, which defines the sensitivity, can be improved by increasing the number of repetitions, but this would require a substantial extension of the analysis time per sample.

### 2.2. Application of ^1^H NMR Spectroscopy to NAD Metabolome Analysis in Human Cell Extracts

To quantify NAD and its major metabolites in extracts of human cells using NMR spectroscopy, first of all, an appropriate extraction protocol has to be established. As described in Materials and Methods, we tested acetonitrile (67%) and methanol (80%) for extraction. Moreover, under these conditions proteins were efficiently precipitated out from the cell extracts. Removing proteins from the sample solution improved the NMR spectrum baseline and reduced the line width and removed signals from the proteins in the range of interest. Further, in order to optimize the dynamic range used, we eliminated strong signals from the solvents by sample lyophilization and dissolving in D2O based NMR buffer.

**Figure 2:**
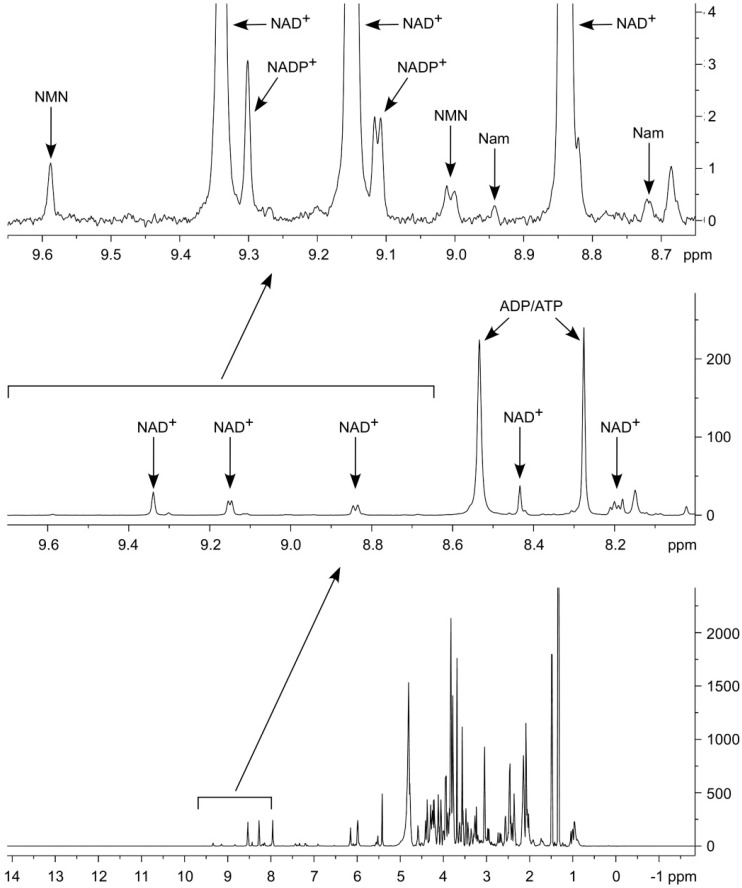
Detection of NAD^+^ and its major intermediates in human cell extracts. Proton nuclear magnetic resonance spectrum of cell extract obtained from HEK293 cells cultivated in the presence of Nam. Spectral region containing peaks corresponding to NAD^+^ and its major intermediates is expanded. The number of scans (nt) was 8192.

In Figure 2, the ^1^H NMR spectrum of metabolites extracted from HEK293 cells using 67% acetonitrile is shown. In the region of chemical shifts 9.6–8.6 ppm (found to be most relevant for NAD and its major metabolites, cf. Figure 1C) we could identify NAD^+^, NADP^+^, NMN and Nam. Importantly, in this area of the spectrum, there are no signals detectable that originate from other compounds in the extract. This is an important advantage for the accurate quantification of the metabolites. The concentrations of the detected compounds (in pmol/mg of protein) are 4.19 ± 0.15 (NAD^+^), 0.42 ± 0.05 (NADP^+^), 0.24 ± 0.04 (NMN), and 0.15 ± 0.03 (Nam). These values are well in the range of those previously reported in the literature [17,18,19,20].

In Figure 2, it is also shown that ATP and ADP were readily detectable in the spectra. However, we could not detect AMP, adenosine or ADP-ribose, suggesting that these adenine derivatives are present only at rather low concentrations in HEK293 cells, given that they were readily detected in standard measurements (Figure S1).

**Figure 3:**
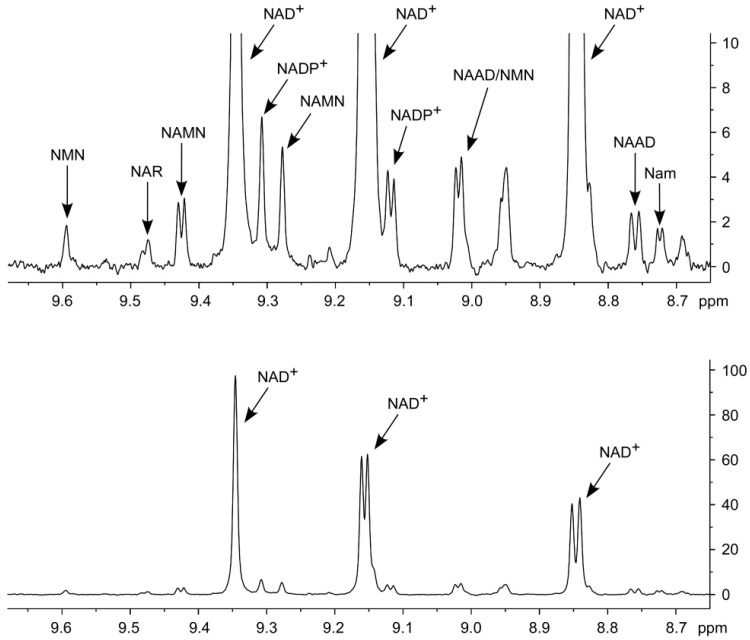
Detection of deamidated NAD^+^ intermediates in human cell extracts. Characteristic region of ^1^H NMR spectrum of cell extract obtained from HEK293 cells cultivated in the presence of Nam, NR and NAR. The upper panel spectrum represents 10× vertically enlarged the lower panel spectrum. nt = 8192.

The cells used for the experiment shown in Figure 2 were grown in standard medium containing 30 µM Nam as the only NAD precursor. Interestingly, when adding 100 µM of NR and NAR to the medium, a significant increase of the amidated metabolites could be detected (Figure 3, Table 2). Moreover, under these conditions, all NA derivatives (NAR, NAMN and NAAD) were detectable and quantifiable (Figure 3, Table 2). 

This finding points to a significant role of the Nam-independent NAD biosynthesis routes. Surprisingly, NR was undetectable in the cell extracts, even when 100 µM of NR were added to the medium. Perhaps, NR is rapidly metabolized to NMN or cleaved to Nam by nucleoside phosphorylase [21,22]. The redox ratio of NAD^+^/NADH is an important parameter for the assessment of cellular activity. We were wondering why the reduced nucleotide was not detected in the cell extracts. However, when cell extraction was conducted using 80% methanol instead of acetonitrile, NADH became clearly detectable (Figure S2). Indeed, NADH oxidation to NAD^+^ is a problem and likely the cause for the absence of NADH in the acetonitrile extracts. However, it is stable in the methanol extracts. 

**Figure 4:**
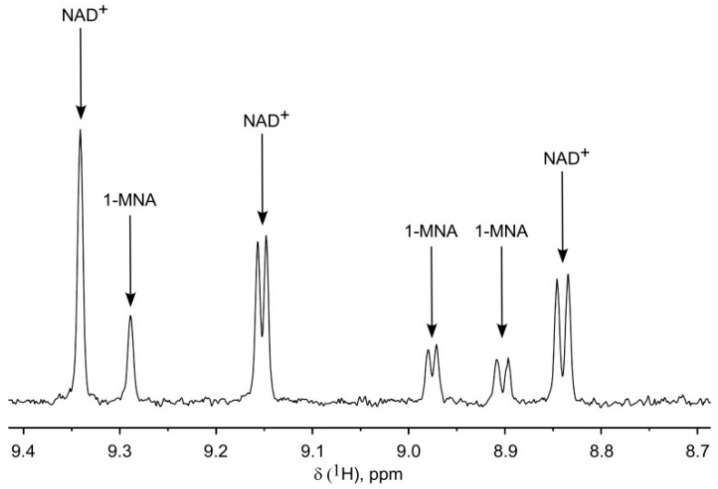
Detection of 1-MNA in human cell extracts. Characteristic region of ^1^H NMR spectrum of cell extract obtained from HeLa cells cultivated in the presence of Nam. nt = 416.

A catabolite of Nam, 1-MNA has recently been shown to have potential regulatory functions [23,24]. While this molecule was readily detectable in the NMR spectrum when a standard compound was used (Figure 1), it was not found in the HEK293 cell extracts. This was not unexpected, because HEK293 cells do not express NNMT, the enzyme that methylates Nam to 1-MNA [25,26]. Indeed, when we extracted and analyzed HeLa cells, which do express NNMT [26], 1-MNA was clearly detectable (Figure 4).

Further, we approved this method for the quantitative analysis of NAD^+^ in human blood cells. Platelets and erythrocytes were isolated from the human whole blood as described in “Materials and Methods” and analyzed by NMR spectroscopy. The only metabolite detectable in the region of chemical shifts 9.6–8.6 ppm was NAD^+^ (Figure S3), and its concentration was estimated as 0.785 ± 0.102 nmol/10^9^ cells for platelets and 2.524 ± 0.164 nmol/10^9^ cells for erythrocytes.

**Figure 5:**
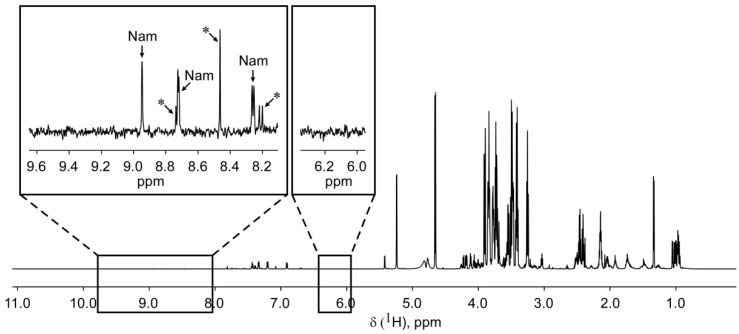
Detection of NAD^+^ intermediates in cell culture medium. Proton nuclear magnetic resonance spectrum of Dulbecco’s Modified Eagle’s Medium (DMEM) supplemented with 10% fetal bovine serum contains Nam as the only NAD^+^ precursor. Two spectral regions containing peaks corresponding to NAD^+^ intermediates are expanded. Asterisks designate unidentified peaks. nt = 256.

Finally, we tested whether the developed method could also be used to determine NAD metabolites in the cell culture medium. Figure 5 shows a ^1^H NMR spectrum obtained from the standard Dulbecco’s Modified Eagle’s Medium (DMEM) medium supplemented with 10% fetal bovine serum and containing only nicotinamide as NAD precursor. As for the cell extracts, the regions of the NMR spectrum relevant for the measurement of NAD metabolites (chemical shifts of 6.0–6.3 and 8.0–9.7 ppm) are almost devoid of signals originating from other compounds present in the culture medium. There are only a few unidentified peaks (marked with an asterisk) that do not coincide with any of the NAD derivatives. These observations indicate that NAD metabolites can, in principle, also be detected and quantified in cell culture medium. In fact, this has recently been shown for the bases (Nam and NA) and nucleosides (NR and NAR) [14]. 

## 3. Materials and Methods

### 3.1. Materials

Unless otherwise specified, all chemicals and reagents were of analytical grade and were purchased from Sigma (St. Louis, MO, USA) and Amresco (Solon, OH, USA). Cell culture reagents were from Gibco (Paisley, UK), Greiner Bio-One (Leipzig, Germany), and Orange Scientific (Braine-l’Alleud, Belgium). High-performance liquid chromatograph grade methanol and acetonitrile were obtained from Merck. The ultrapure water was obtained from a Milli-Q Synthesis purification system (Millipore, Darmstadt, Germany). Nicotinamide riboside was synthesized as reported previously [27].

### 3.2. Large Scale Synthesis of NAR

To a dry round-bottom flask was added nicotinic acid (40 g, 324.9 mmol, 1.0 equiv.), followed by HMDS (200 g, 1239.2 mmol, 3.8 equiv.) and a catalytic amount of ammonium sulphate (1% mol equiv.). The suspension was then heated to reflux under an atmosphere of nitrogen for 12 h. The solution was cooled to room temperature, and the excess HMDS was removed under reduced pressure. The gummy oil was then resuspended in freshly distilled dichloroethane (150 mL), followed by the addition of 1,2,3,5-tetra-*O*-acetyl-β-d-ribofuranose (103 g, 322.6 mmol, 1.0 eq) and TMSOTf (58 mL, 322.6 mmol, 1.0 equiv.). The solution was heated to 40 °C and left stirring overnight under an atmosphere of nitrogen. After ^1^H NMR analysis indicated that the reaction had reached completion, the solution was allowed to cool to room temperature. With intensive stirring, 100 mL of distilled water was added followed by the rapid addition of a saturated NaHCO_3_ solution (approximately 50 mL). The pH was adjusted to approximately 6, and the organic phase was separated, then the aqueous layer was washed three additional times with dichloromethane (100 mL). The aqueous layer was then frozen and freeze-dried to yield crude nicotinic acid riboside triacetate (NAR-TA) as an off-white solid which was used in the subsequent step without any further purification. In a glass pressure tube, the crude NAR-TA was suspended into methanol, and ammonia gas was bubbled into the solution for 10 min with the temperature held at −78 °C. The tube was then sealed and stored at −20 °C for 4 days, after which the solution was concentrated under reduced pressure. The crude was then re-solubilized into methanol and an equivalent volume of acetone was added, causing a thick precipitate to occur. The precipitate was then filtered under reduced pressure and washed an additional five times with cold methanol to yield nicotinic acid riboside as a free-flowing orange powder in 74% yield. ^1^H NMR (400 MHz, D_2_O): *δ* ppm 9.33 (br s, 1H, aromatic), 9.02 (d, *J* = 6.3 Hz, 1H, aromatic), 8.81 (dt, *J* = 8.0, 1.3 Hz, 1H, aromatic), 8.06 (dd, *J* = 8.0, 6.3 Hz, 1H, aromatic), 6.09 (d, *J* = 4.8 Hz, 1H, H-1 (anomeric)), 4.37 (dd, *J* = 4.8, 4.5 Hz, 1H, H-2), 4.33–4.36 (m, 1H, H-4), 4.23 (t, *J* = 4.5 Hz, 1H, H-3), 3.91 (ABX, *J*A,A′ = 12.9 Hz, *J*A,B = 3.9 Hz, 1H, H-5), 3.78 (ABX, *J*A,A′ = 12.9 Hz, *J*A,B = 2.9 Hz, 1H, H-5′). 13C NMR (100 MHz, D2O): *δ* ppm 167.5 (COOH), 146.9, 141.3, 140.9, 137.3, 127.9 (aromatic), 99.6 (C-1 (anomeric)), 87.6 (C-4), 77.5 (C-2), 70.0 (C-3), 60.4 (C-5). HRMS (ES, M + H^+^) calculated 256.0821 for C_11_H_13_NO_6_, found 256.0818.

### 3.3. Cell Culture

The HEK293 and HeLa cells (obtained from American Type Culture Collection (ATCC)) were cultivated in DMEM supplemented with 10% fetal bovine serum, 2 mM glutamine, and penicillin/streptomycin. The cells were cultured at 37 °C in a humidified atmosphere of 5% CO_2_. Nicotinamide riboside and NAR (100 µM) were added to the medium as indicated. 

### 3.4. Platelets and Erythrocytes Isolation from the Human Whole Blood

Blood was collected from healthy volunteers after obtaining informed consent and according to the Declaration of Helsinki. Our studies with human platelets and erythrocytes were approved and reconfirmed (protocol #7 from 10 February 2014) by the local Ethical Committee of the Sechenov Institute of Evolutionary Physiology and Biochemistry. Blood was collected from antecubital area into 1/7 volume of ACD solution (12 mM citric acid, 15 mM sodium citrate, 25 mM d-glucose and 2 µM EGTA, final concentrations). Platelet rich plasma (PRP) was obtained by 5 min centrifugation at 330× *g*. Platelet rich plasma was centrifuged for 10 min at 430× *g*, then the pelleted platelets were washed once in CGS buffer (120 mM sodium chloride, 12.9 mM trisodium citrate, 30 mM d-glucose, pH 6.5), and resuspended in HEPES buffer (150 mM sodium chloride, 5 mM potassium chloride, 1 mM magnesium chloride, 10 mM d-glucose, 10 mM HEPES, pH 7.4). Red blood cells were collected after separation of PRP and washed thrice with HEPES buffer. 1 × 10^9^ platelets and 2 × 10^9^ erythrocytes were used for NMR analysis. Platelet and erythrocyte concentrations were controlled by Medonic M-20 (Boule Medical, Spånga, Sweden).

### 3.5. NMR Sample Preparation

For metabolite extraction, HEK293 and HeLa cells (2 × 10^7^) grown on 100 mm cell culture plates were washed twice with ice-cold (4 °C) phosphate-buffered saline (PBS) and put on ice. Following addition of either 67% acetonitrile or 80% methanol, the cells were kept on ice for 30 min. Thereafter, the cells were scraped off and centrifuged at 15,000× *g* for 30 min at 4 °C. The obtained pellets were used for protein determination using BCA Protein Assay kit (Thermo Fisher Scientific, Rockford, IL, USA). Metabolites from 1 × 10^9^ platelets and 2 × 10^9^ erythrocytes were extracted with methanol as described above. Cell extracts were lyophilized and then resuspended in DBP buffer, D_2_O-based buffer containing 50 mM NaPi (pH 6.5) and 1 mM sucrose as a chemical shift reference (*δ*(1H), 5.42 ppm) and internal standard for quantification. To remove oxygen, the samples were kept under vacuum (80 mm Hg) for 10 min with occasional agitation. Samples were stored at −80 °C until NMR analysis. 

Cell culture medium was also collected and metabolites extracted by adding 2 volumes of acetonitrile and then treated in the same way as the cell extracts.

The 100 µM standard solutions of Nam, NA, NR, NAR, NMN, NAMN, NAD^+^, NAAD, NADP^+^, NADH, NADPH, and 1-MNA were prepared using DBP buffer. Samples were stored at −80 °C until NMR analysis.

### 3.6. NMR Analysis and Quantification

All NMR experiments were performed at 25 °C using a Varian DirectDrive NMR System 700-MHz spectrometer (Varian Inc., Palo Alto, CA, USA) equipped with a 5-mm z-gradient salt-tolerant probe. The one-pulse sequence with the suppression of solvent signal by presaturation was used. Each FID was acquired during 3 s with sweep width of 11,160 Hz, 2 s relaxation delay, and 32 K time domain complex points. Different numbers of transitions (nt = 128 ÷ 8192) were collected. Data were acquired using VNMRJ 4.2 (Agilent Technologies, Santa Clara, CA, USA). and then analyzed by Mestrelab Mnova (version 12; Mestrelab, Santiago de Compostela, Spain). The FIDs were processed with line-broadening of 1.5 Hz and zero-filling up to 64 K points before Fourier transformation. Basic processing of spectra—phasing, baseline correction and spectra lines assignment of metabolites—was performed in batch mode with subsequent visual control of each spectrum. Spectra were referenced to the 1H signal of sucrose at *δ* = 5.42 ppm.

Longitudinal relaxation times, *T*_1_, were measured by the inversion recovery pulse sequence from the Agilent Pulse sequence library in 1 mM metabolite solutions in DBP buffer. Phase cycling was used to eliminate residual pulse length and phase errors. Up to 8 scans were accumulated for each value of interpulse delay (*τ*) to improve signal-to-noise ratio. The relaxation experiment comprised 11 *τ* values in the interval (0.05–5.0) × *T*_1_. The relaxation times were evaluated from exponential nonlinear fits to the data points using VNMRJ 4.2 software.

For quantitative analysis, only those signals were used that did not overlap with others. Each peak of the assigned multiplet signal was fitted with the Loretz–Gauss function, and the total signal intensity was determined as the sum of the integrals of the peaks. For the fitting procedure and signal assignment the Mestrelab Mnova program was used. Metabolite concentration *C* was calculated according to Equation (1):

(1) $$C=\frac{\frac{1}{n}\sum_{i=1}^{n}\alpha_{i}I_{i}^{}}{I_{0}}$$

where *I*_0_ is the intensity of 1H proton of glucose moiety in sucrose (1 mM) at *δ* = 5.42 ppm, *I_i_* is the intensity of *i*th multiplet signal, *α_i_*, the longitudinal relaxation correction coefficient for *i*th multiplet, is defined according to Equation (2):

(2) $$\alpha_{i}=1-\exp\left\{-\frac{t_{0}}{T_{1i}}\right\}$$

where *T*_1*i*_ is the longitudinal relaxation time of *i*th proton and *t*_0_ is the repetition time equal to the sum of the acquisition time and the relaxation delay time. The presented quantitative data were obtained by normalizing the measured metabolite contents to the protein amount in the sample.

## 4. Conclusions

We have developed an NMR-based experimental approach suitable for the quantitative analysis of the NAD metabolome in human cells. The basic NMR characteristics (relaxation time, chemical shifts) were determined for all intermediates of NAD biosynthesis. Moreover, the parameters for recording of the NMR spectra as well as the conditions for sample preparation were critically assessed and optimized. Our results show that NMR represents a powerful method for the quantitative evaluation of the NAD metabolome in biological samples which in some instances may be superior to mass spectrometry-based methods, in particular, with regard to relative quantification within a given sample.

## Figures and Tables

**Table 1:** Proton nuclear magnetic resonance (^1^H NMR) spectral parameters of NAD and its major intermediates.

Metabolite	*δ*, ppm	M, Class	J’s, Hz	*T*_1_, s
Nam	8.94	s	/	13.73
	8.71	d	5.01	7.97
	8.25	d	7.97	8.52
	7.60	dd	4.93, 7.99	6.29
NR	9.64	s	/	3.02
	9.31	d	6.18	2.21
	9.02	d	8.03	5.29
	8.31	t	7.11, 7.11	2.22
	6.29	d	4.39	1.89
NMN	9.60	s	/	3.16
	9.34	d	6.26	2.56
	9.00	d	8.03	4.68
	8.32	t	7.11, 7.11	2.97
	6.20	d	5.83	2.27
NAD^+^	9.34	s	/	2.32
	9.15	d	6.22	1.76
	8.84	d	8.04	3.99
	8.43	s	/	2.84
	8.20	t	7.19, 7.19	2.29
	8.18	s	/	9.73
	6.09	d	5.44	1.8
	6.04	d	5.94	4.09
NA	8.94	d	2.14	12.23
	8.61	d	4.99	9.41
	8.25	d	7.9	10
	7.52	dd	4.90, 7.91	6.44
NAR	9.47	s	/	2.93
	9.16	d	6.2	2.06
	8.95	d	7.91	4.92
	8.20	t	7.03, 7.03	2.38
	6.23	d	4.68	1.89
NAMN	9.44	d	6.23	1.92
	9.26	s	/	2.09
	8.94	d	7.88	4.11
	8.27	t	7.07, 7.07	2.25
	6.19	d	5.37	1.75
NAAD	9.13	s	/	2.1
	9.02	d	6.22	1.97
	8.75	d	7.87	3.96
	8.44	s	/	2.67
	8.15	s	/	8.99
	8.06	t	7.04, 7.04	2.18
	6.05	t	5.36, 5.36	2.92
NADH	8.49	s	/	2.61
	8.25	s	/	7.79
	6.95	s	/	1.79
	6.13	d	5.51	3.78
	5.99	d	8.04	1.50
NADP^+^	9.30	s	/	2.4
	9.12	d	6.29	1.82
	8.83	d	8.09	4.09
	8.42	s	/	2.92
	8.19	m	/	2.33
	8.16	s	/	9.6
	6.12	d	5.1	3.95
	6.04	d	5.62	1.75
NADPH	8.48	s	/	2.84
	8.25	s	/	8.05
	6.94	d	1.53	1.92
	6.23	d	4.81	3.8
	5.97	d	8.53	1.75
1-MNA	9.29	s	/	6.98
	8.98	d	6.08	5.8
	8.90	d	8.20	7.17
	8.19	dd	6.16, 8.15	5.0

Metabolites were dissolved in 50 mM sodium phosphate buffer in D_2_O (pH 6.5). *δ*—chemical shift (ppm) in relation to sucrose signal (*δ*(1H), 5.42 ppm), M—multiplicity ((s) singlet, (d) doublet, (t) triplet, (dd) double doublet), J’s—coupling constants (Hz), *T*_1_—longitudinal relaxation time (s).

**Table 2:** The intracellular NAD^+^ and its major intermediates content (nmol/mg protein) in HEK293 cells.

Metabolite	Untreated	Treated with NR and NAR
NAD^+^	4.19 ± 0.15	8.69 ± 0.69
NMN	0.24 ± 0.04	0.27 ± 0.06
NR	nd *	nd
Nam	0.15 ± 0.03	0.54 ± 0.12
NADP^+^	0.42 ± 0.05	0.55 ± 0.03
NAAD	nd	0.62 ± 0.04
NAMN	nd	0.42 ± 0.05
NAR	nd	0.09

* not detected.

## Supplementary Materials

Supplementary Materials can be found at http://www.mdpi.com/1422-0067/19/12/3906/s1.
