# Supplementary material for: NAD Metabolome Analysis in Human Cells Using 1H NMR Spectroscopy
Source: Int J Mol Sci. 2018 Dec 6;19(12):3906. doi: 10.3390/ijms19123906 (PMC6321329; doi:10.3390/ijms19123906)
Supplement: Supplementary file 1 [file ijms-19-03906-s001.pdf]

Supplementary Data

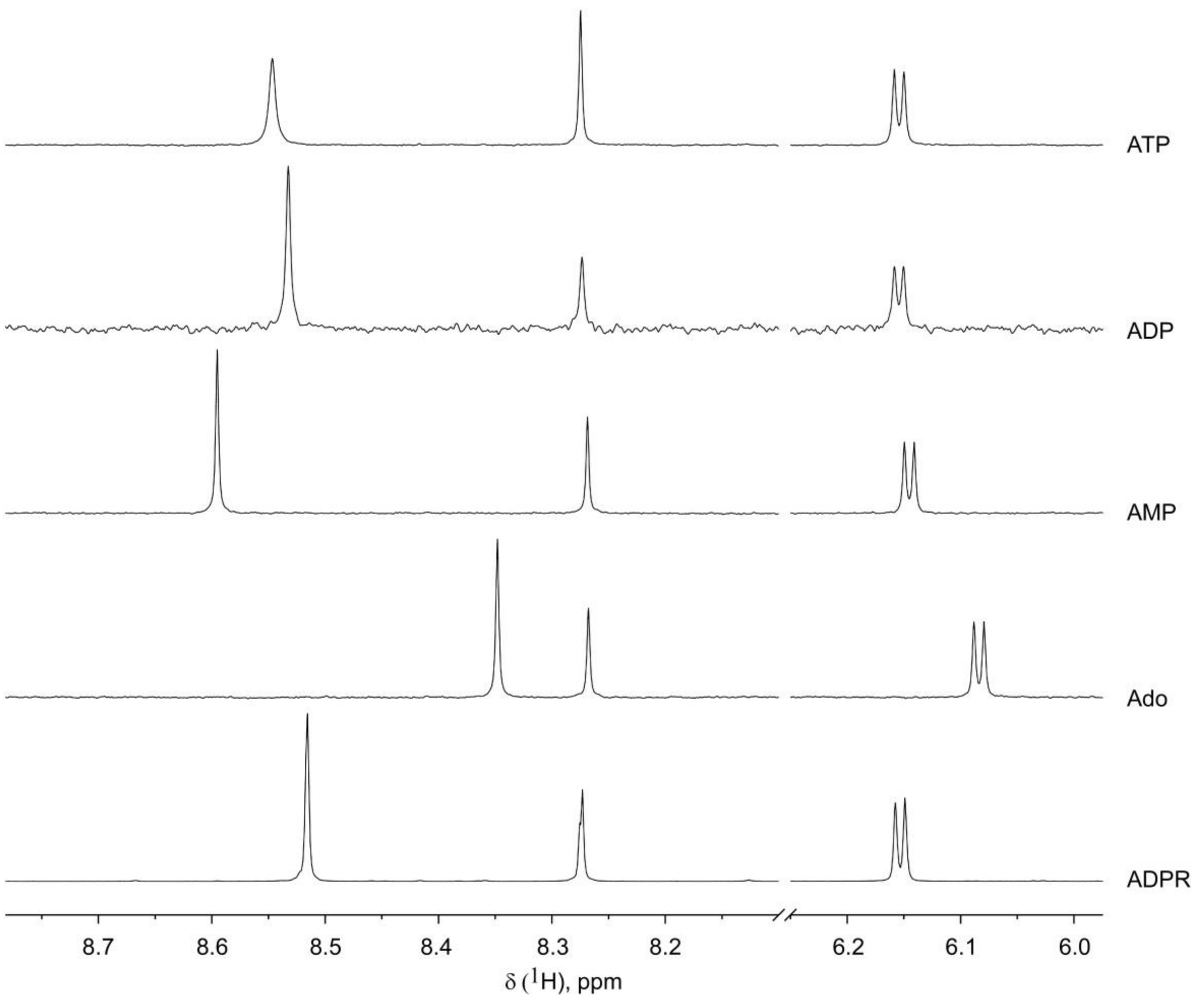

Figure S1. 700 MHz ^{1}H NMR spectra of adenosine-containing compounds. Metabolites were dissolved in 50 mM sodium phosphate buffer in D_2O (pH 6.5) and analyzed by NMR spectroscopy.

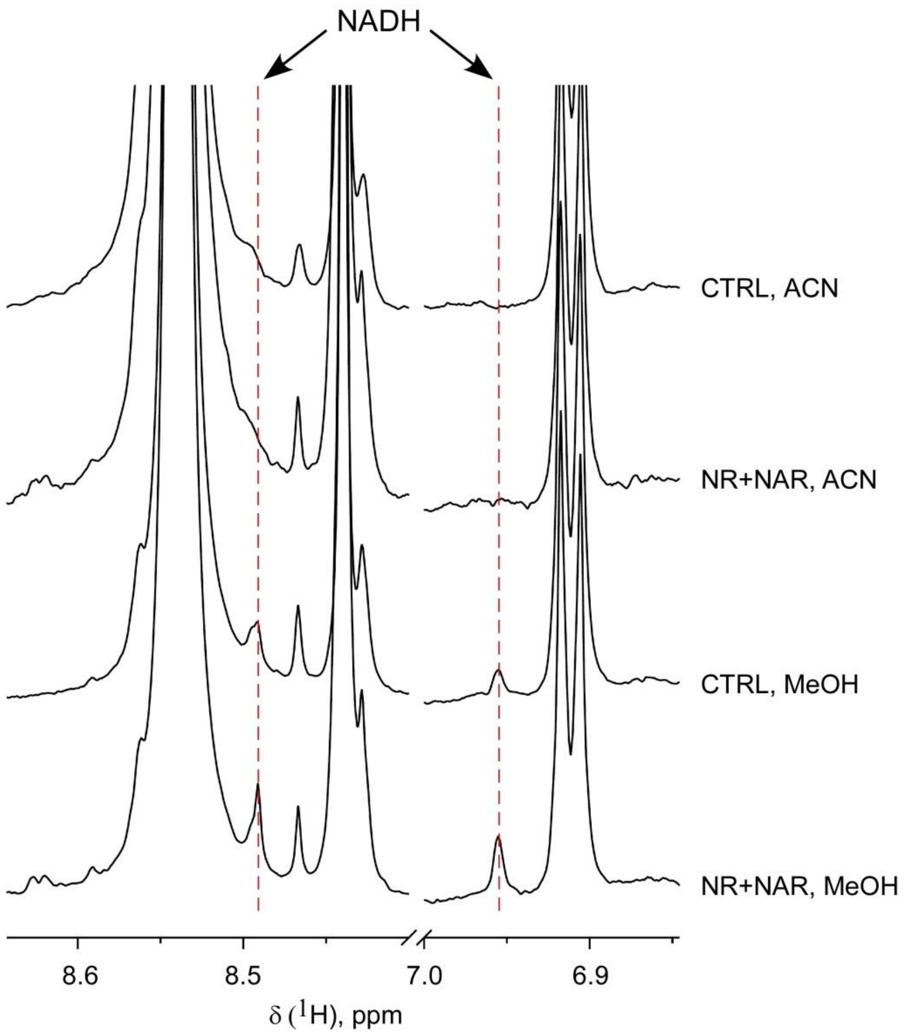

Figure S2. Detection of NADH in human cell extracts. Characteristic region of ^{1}H NMR spectra of cell extracts obtained from HEK293 cells cultivated in the presence of Nam (CTRL) or additionally with NR and NAR. Cell extracts were prepared using acetonitrile (ACN) or methanol (MeOH). nt = 8192.

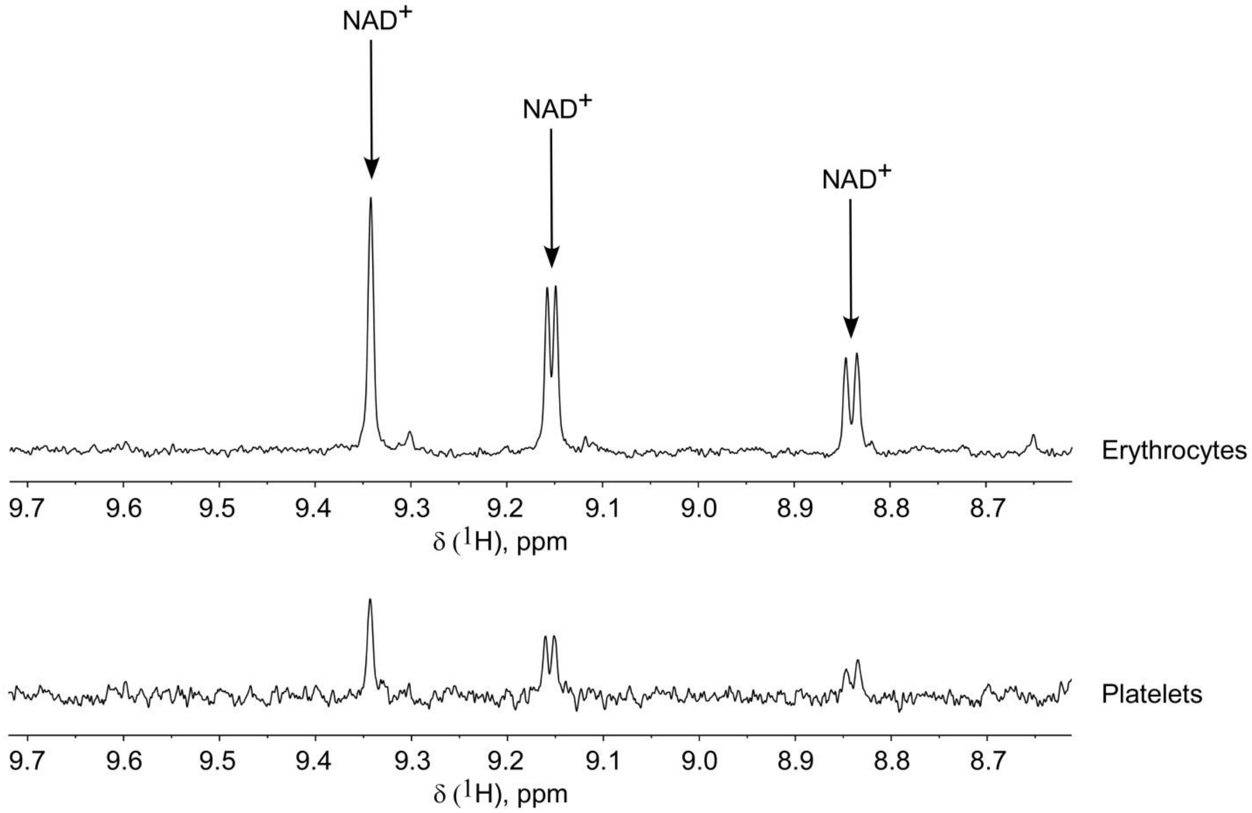

Figure S3. Detection of NAD^+ in human blood cells. Characteristic region of ^1H NMR spectrum of cell extracts obtained from isolated human erythrocytes and platelets. nt = 10240.
